# The Perception of Teaching, Learning Styles and Commitment to Learning and Their Influence on the Practice of Physical Activity and Eating Habits Related to the Mediterranean Diet in Physical Education Students

**DOI:** 10.3389/fpsyg.2022.927667

**Published:** 2022-06-22

**Authors:** Carmen Fernandez-Ortega, Jeronimo González-Bernal, Sergio Gonzalez-Bernal, Ruben Trigueros, José M. Aguilar-Parra, Luis A. Minguez-Minguez, Ana I. Obregon, Raquel De La Fuente Anuncibay

**Affiliations:** ^1^Department of Psychology, University of Burgos, Burgos, Spain; ^2^Department of Psychology, University of Almería, Almería, Spain; ^3^Department of Mathematics, University of Burgos, Burgos, Spain

**Keywords:** teaching, physical education, critical thinking, metacognitive strategies, healthy lifestyle habits

## Abstract

Childhood obesity, linked to a sedentary lifestyle and an unbalanced diet, is one of the main problems in today’s Western societies. In this sense, the aim of the study was to analyze students’ perceived satisfaction in physical education classes with learning strategies and engagement in learning and critical thinking as determinants of healthy lifestyle habits. The study involved 2,439 high school students aged 12–18 years (*M* = 14.66, *SD* = 1.78). Structural equation modeling was conducted to analyze the predictive relationships between the study variables. The results showed that teaching, teaching mastery, and cognitive development are precursors to deep thinking on the part of students, indicators of the adoption of healthy lifestyle habits. These results reflect the importance of the methodology adopted by the teacher in order to positively influence the students’ habits.

## Introduction

There is currently a growing concern about unhealthy eating habits and lack of physical activity in the adolescent population (Jonsson et al., 2017). In this regard, a recent survey conducted in Spain revealed that 5.1% of adolescents suffer from obesity and 21.4% are overweight (Moreno et al., 2020). This is due to the abandonment of the Mediterranean diet, with adolescents’ diets being unbalanced and tending toward a high fat intake (35–50% of total calorie content), with a low polyunsaturated/saturated fatty acid index. In addition, the lack of physical activity among adolescents, with only 39.5% of young people engaging in regular physical activity, increases the health risk situation of young people, who are more prone to suffer from coronary heart disease, hepatitis, metabolic diseases, etc. (Tucker et al., 2011). Therefore, Physical Education (PE) classes have been shown to be a key factor in raising awareness and consolidating healthy habits in adolescents (Dudley et al., 2011; Trigueros et al., 2019a), both in terms of sports practice and the benefits of eating a balanced diet, given their ability to generalize beyond the academic context (Hagger and Chatzisarantis, 2007; Ward, 2013). Furthermore, physical activity is one of the main protectors of childhood obesity (Heerman et al., 2022), and its benefits in improving health have been documented (Janssen et al., 2011; Sigmund et al., 2012), which is why it has been repeatedly included in the academic curriculum in different European countries (Eurydice, 2013).

Studies to date have shown that the motivational climate generated by the teacher and classmates has a significant influence on student engagement and participation during PE lessons (Newland et al., 2017; Zach et al., 2020). However, it is necessary to take into consideration students’ perceived satisfaction with the teacher’s teaching methodology and its influence on the students’ learning process (Kosiba et al., 2019). In this sense, a study by Trigueros et al. (2019b) highlights the presence of three essential components with teaching satisfaction: teaching, which refers to the student’s assessment of the pedagogical quality of the teaching staff, their attitude and their explanations; cognitive development, which refers to satisfaction with the perceived improvement of learning, both in mental strategies and basic concepts of the subject; and mastery teaching, which refers to learning and improvements in the mastery of physical and motor skills related to sports practice. In short, satisfaction would reflect the effect that the context, methodology and experiences in the PE class have on the students (Ramírez et al., 2019; Korina et al., 2021). This construct has been closely related to performance as it involves procedural learning aimed at perfecting physical skills, as well as effort, the desire to excel, motivation for the activity, the importance given to PE and the intention to remain physically active (Ruiz-Juan et al., 2010; Baena-Extremera et al., 2012), reflecting the effect that the environments, methods and experiences in the PE class have on students (Cid et al., 2019).

On the other hand, it is essential to consider the importance of learning strategies, critical thinking and commitment to learning toward the achievement of academic goals (Ibrahim et al., 2017). In this sense, it is important to encourage students’ autonomy in planning and managing their own learning in order to become aware of their own mistakes in their teaching-learning process, which prevent them from achieving academic goals (Colomer et al., 2018). In addition, young people in today’s society are subjected to a constant flow of information, most of the time untruthful, so it is important to develop in our students an open mind, but with a deep critical spirit, being necessary the acquisition of higher levels of thinking (Radulović and Stanèić, 2017). However, for students to be able to manage their learning and to develop a critical spirit, engagement in learning is an important mediator in determining learning outcomes, as greater student engagement can improve academic outcomes and commitment to set goals (Wu et al., 2020).

These three psychological elements related to the management and organization of information can have a substantial influence in facilitating the study and/or predicting the consolidation of future behaviors related to a balanced diet, typical of the Mediterranean diet, and the practice of physical activity, which are the one of didactic objective of the area of PE according to the Secondary Education Curriculum (Real Decreto 217/2022). In this sense, the use of deep learning strategies (metacognition) requires effort on the part of the learner that not only means commitment to the goal, but also regulates the continued use that can be maintained over time (Ulstad et al., 2016). Similarly, students’ engagement and involvement in classes is positively associated with intrinsic motivation and leads to greater dedication and enjoyment toward physical activity practice (Gorely et al., 2009). In relation to adherence to these healthy habits and PE classes, previous research studies show that the PE teacher himself, in addition to the didactic contents of the subject itself, are an important element in achieving these habits (Tobar et al., 2019). It is highlighted that the content blocks of games and sports, health, and physical fitness play a fundamental role in students’ likes and desires for physical activity in PE classes. In most cases, this is reflected in pupils’ lifestyles. That is, their attachment to the PE subject and their subsequent success is directly related to their engagement in physical activity outside the classroom (Valencia-Peris and Mora, 2018). Furthermore, clear improvements are observed in students’ self-esteem, self-concept and perceptions, while enhancing values related to effort, respect and teamwork, key values for the promotion of physical activity and health at school. Similarly, nutritional habits constitute one of the objectives of the new educational law in Spain (*LOMLOE; Ley Orgánica por la que se Modifica la Ley Orgánica 2/2006*), which is related to the acceptance of one’s own body, that of others and using PE as a way of consolidating healthy eating habits and the practice of physical activity. In this sense, Vaquero-Solís et al. (2021) highlighted that, in addition to adequate levels of physical activity, aspects such as regular consumption of fruit and vegetables, fish and healthy breakfast habits, typical of the Mediterranean diet, should be considered as protective factors in relation to health. However, content related to food is hardly worked on in the area of PE, despite the fact that, as detailed above, it is an objective of the area.

Thus, the aim of this study is to analyze students’ perceived satisfaction in PE classes with strategies and commitment to learning and critical thinking as determinants of healthy lifestyle habits. To this end, the following hypotheses are put forward: (1) teaching, mastery teaching and cognitive development will correlate positively with each other; (2) teaching, mastery teaching and cognitive development will be positively related to metacognitive strategies, engagement in learning and critical thinking; (3) engagement in learning will correlate positively with critical thinking and metacognitive strategies; (4) metacognitive strategies, engagement in learning and critical thinking will correlate positively with physical activity and healthy eating related to the Mediterranean diet.

## Materials and Methods

### Participants

The secondary school students who participated in the study were 2,439, of which 1,331 were boys and 1,108 girls. These students were studying in different schools in the provinces of Almeria and Granada.

The age of the participants ranged from 12 to 18 years (*M* = 14.66; *SD* = 1.78). 54.44% were in Compulsory Secondary Education and 45.56% were in Post-Compulsory Secondary Education.

The sampling method for the student population was non-probabilistic inferential, based on those educational centers to which access was available. The criteria for participation in the study were the submission of a signed authorization from the parents or legal guardians and the full completion of each of the questionnaires described below.

### Measurement

#### Perceived Structured Physical Education Teaching Environment

The scale used was the Spanish version of the Physical Activity Class Satisfaction Questionnaire (Cunningham, 2007) by Sicilia et al. (2014). The scale is made up of 9 factors and 45 items. However, to measure students’ satisfaction with theoretical knowledge, learning skills and teaching methodology, only three factors were used: cognitive development, teaching mastery and teaching. The Likert scale that students had to fill in ranged from 1 (strongly disagree) to 8 (strongly agree).

#### Metacognitive Strategies and Critical Thinking

The Spanish version of the Motivated Strategies for Learning Questionnaire (MSLQ; Pintrich et al., 1993) by Roces et al. (1995) was used. This questionnaire is made up of 81 items distributed among 15 factors. However, only the 12 items referring to metacognition strategies and the 5 critical thinking items were used. Students were asked to respond on a Likert scale ranging from 1 (not true at all) to 5 (completely true).

#### Engagement With Learning

The Spanish version of the Achievement Motivation in Physical Education Test (Nishida, 1988) of Ruiz et al. (2004) was used. This questionnaire is made up of 32 items spread over 4 dimensions. However, only the 9 items referring to engagement with learning were used. The Likert scale that students had to fill in ranged from 1 = strongly disagree to 5 = strongly agree.

#### Balanced Diet

The Spanish version of the scale linked to the Mediterranean diet (Serra-Majem et al., 2004) was used. This scale consists of 16 items, with an overall score ranging from 0 to 12. Items denoting a negative connotation with regard to the Mediterranean diet were assigned a value of -1, and those with a positive aspect were assigned a value of + 1.

#### Intentionality of Being Physically Active

The Spanish version of the Intention to be physically active scale (Hein et al., 2004) by Moreno et al. (2007) was used. This scale consists of five items to measure a single factor. The items are preceded by the phrase “Regarding your intention to practice some physical/sports activity.” The Likert scale that students had to fill in ranged from 1 (totally disagree) to 5 (totally agree).

### Procedure

To initiate the study, it was necessary to establish contact with the management teams of several educational centers so that they would grant us their authorization to access the students. Both the students and the management team were explained to them and any doubts about the study were resolved.

Those students who wished to participate voluntarily in the study were asked to sign an authorization form signed by their parents or legal guardians. After obtaining permission from the management team, the questionnaires were administered. The students completed the questionnaire individually with pen and paper at the beginning of PE lessons, stressing the anonymity of their answers and respect for all ethical procedures. During this phase, a member of the research group was present to answer any questions that might arise. The estimated time to complete the questionnaires was around 20 min.

The study obtained the approval of the bioethics committee of the University of Almería (Ref. UALBIO 2020/014) and respected the procedures established by the Declaration of Helsinki.

### Data Analysis

The analyses used in the present study were descriptive statistics, represented by the mean, standard deviation and bivariate correlations. In addition, Cronbach’s alpha reliability analyses were calculated. For each of these analyses, the SPSS 25 statistical package was used.

To analyze the objective and hypotheses of the study, a hypothesized model was established using structural equation modeling (SEM). For this purpose, the maximum likelihood method was used, which is the most appropriate in studies using Likert scales as it takes into account the non-normal distribution of the data (Beauducel and Herzberg, 2006). Furthermore, in the SEM model, 95% bias-corrected bootstrap CIs (95% CIBC) were calculated with a bootstrapping of 6,000 interactions (Hayes and Scharkow, 2013).

The model fit indices were used to define good models: the chi-square/degree freedom, the Root Mean Square Error of Approximation (RMSEA) with its 90% confidence interval (CI), the Comparative Fit Index (CFI), Incremental Fit Index (IFI) and Tucker–Lewis Index (TLI). The adjustment rates taken into account for the previous CFAs and SEM were those considered by Hair et al. (2006) (Table 1).

**TABLE 1:** Adjustment indexes.

Estadístics	Good indexes
Chi square/degree freedom	Between 2 and 3
Comparative fit index (CFI)	Greater than 0.95
Incremental fit index (IFI)	Greater than 0.95
Tucker lewis index (TLI)	Greater than 0.95
Root mean square error of approximation (RMSEA) y su intervalo de confianza al 90%	Equal or less than 0.06
Standardized root mean square residual (SRMR)	Equal or less than 0.08

It should be noted that these adjustment indices should be interpreted with caution, as they prove to be too stringent or too complicated to achieve in complex models (Marsh et al., 2004).

## Results

### Preliminary Analysis

The bivariate correlations, as shown in Table 2, were positive between each of the study variables. Finally, the reliability analysis showed a score above 0.70, so each of the factors were considered reliable (Taber, 2018).

**TABLE 2:** Descriptive statistics and correlations between all variables.

Factors	*M*	*SD*	α	1	2	3	4	5	6	7	8
1. Cognitive development	5.65	0.93	0.80	−	0.31**	0.33**	0.54***	0.35**	0.37***	0.45***	0.31**
2. Mastery teaching	6.04	0.68	0.83		−	0.49**	0.48***	0.42**	0.42**	0.31**	0.30*
3. Teaching	6.18	0.71	0.85			−	0.56**	0.36**	0.35***	0.44***	0.26***
4. Metacognitive strategies	3.63	1.12	0.82				−	0.58***	0.46**	0.32**	0.22*
5. Learning engagement	3.83	1.07	0.86					−	0.31**	0.56***	0.31***
6. Critical thinking	3.53	1.14	0.83						−	0.66***	0.59**
7. Intention to be Physically active	5.35	0.78	0.80							−	0.64***
8. Mediterranean diet	8.22	0.69	0.79								−

***p < 0.001; **p < 0.01; *p < 0.05.

### Structural Equation Model

**FIGURE 1:**
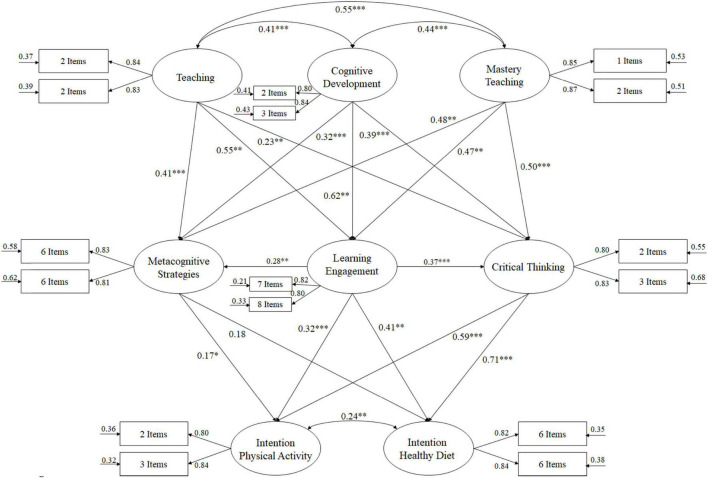
Structural equational modeling. ^***^p < 0.001; ^**^p < 0.01; *p < 0.05.

The fit indices of the hypothesized model, through structural equation analysis (Figure 1), to analyze the predictive relationships were adequate: χ^2^(84, *N* = 3,415) = 254.38, χ^2^/df = 3.03, *p* < 0.001, IFI = 0.94, TLI = 0.94, CFI = 0.94, RMSEA = 0.061 (CI 90% = 0.055–0.065), SRMR = 0.041. These indices reflect that the model has had an acceptable fit and should therefore be considered adequate. Standardized regression tests were used to analyze the relationships between the study variables.

The relationships obtained between the different factors that make up the model (Figure 1) are described as follows:

a) The correlations were positive, being β = 0.41 (*p* < 0.001) between teaching and cognitive development; β = 0.55 (*p* < 0.001) between teaching and mastery experiences; β = 0.44 (*p* < 0.001) between cognitive development and mastery experiences.
b) The relationship between teaching and metacognitive strategies (β = 0.41, *p* < 0.001), engagement in learning (β = 0.55, *p* < 0.01) and critical thinking (β = 0.23, *p* < 0.01), was positive.
c) The relationship between cognitive development and metacognitive strategies (β = 0.32, *p* < 0.001), engagement in learning (β = 0.62, *p* < 0.01) and critical thinking (β = 0.39, *p* < 0.001), was positive.
d) The relationship between mastery experiences and metacognitive strategies (β = 0.48, *p* < 0.01), engagement in learning (β = 0.47, *p* < 0.01) and critical thinking (β = 0.50, *p* < 0.001) was positive.
e) The relationship between commitment to learning and metacognitive strategies (β = 0.28, *p* < 0.01) and critical thinking (β = 0.37, *p* < 0.001) was positive.
f) The relationship between metacognitive strategies and intention to be physically active (β = 0.17, *p* < 0.05) and Mediterranean diet (β = 0.18, *p* < 0.06) was positive.
g) The relationship between engagement in learning and intention to be physically active (β = 0.32, *p* < 0.001) and Mediterranean diet (β = 0.41, *p* < 0.01) was positive.
g) The relationship between critical thinking and intention to be physically active (β = 0.59, *p* < 0.001) and Mediterranean diet (β = 0.71, *p* < 0.001) was positive.
h) The correlation was positive between intention to be physically active and intention to maintain a healthy diet (β = 0.24, *p* < 0.01).

## Discussion

The aim of the present study was to analyze the satisfaction perceived by students in PE classes on strategies and commitment to learning and critical thinking as determinants of PE academic objectives related to healthy lifestyle habits related to the practice of physical activity and balanced eating. This study presents for the first time a direct relationship between students’ perceived mastery experiences of their own learning processes present during PE lessons and the achievement of academic goals related to the adoption of healthy lifestyle habits. So far, studies have focused on students’ motivation toward PE classes (Behzadnia and Ryan, 2018; Bechter et al., 2018; Trigueros et al., 2019b; Mastagli et al., 2021) and its relationship with respect to the adoption of future behaviors. In this sense, more than motivation and its relationship with adaptive behaviors, it is necessary to analyze how students process and understand the information that comes to them during PE classes in order to adapt methodologies to make it easier to understand and assimilate the information and the importance of achieving academic goals (Goodyear and Dudley, 2015; Mannino et al., 2019).

Results have revealed how cognitive development, mastery experiences and teaching directly influence metacognitive strategies, engagement in learning and critical thinking. These results cannot be contrasted with previous studies in either the setting of the PE classroom or the educational context. However, a study by Ferriz et al. (2016) and Trigueros et al. (2019b) revealed that cognitive development, mastery experiences and teaching were predictors of the satisfaction of basic psychological needs (competence, relatedness to others, autonomy, and novelty). These psychological needs have in turn been linked to students’ search for new experiences culminating in the development of critical thinking, metacognition and students’ engagement during lessons due to the development of their motor skills (Zhang et al., 2019). Similarly, a study by Ulstad et al. (2016) showed how teachers’ autonomy support was important in meeting basic psychological needs that mediated their relationships with motivational constructs. It was also important for teachers to encourage students to use learning strategies and to teach them how to use them in order to participate and perform better in school, achieving academic goals. Therefore, this study highlights the need to work on aspects that will subsequently influence student satisfaction, such as the structure of lessons, the promotion of innovative learning or the need to provide an appropriate adjustment to the learning pace of each student. Thus, teachers should foster student motivation through the development of a sense of competence, positive interpersonal relationships, or the design of innovative activities (Casey and MacPhail, 2018; Cheon et al., 2019; Trigueros and Navarro, 2019). In addition, content should have maximum meaning and significance for students and should be appropriate to their motivational needs and interests, in order to achieve greater commitment and involvement in learning (Moreno-Doña et al., 2016).

In addition, results showed that metacognitive strategies, engagement in learning and critical thinking were positively related to intention to be physically active and the consumption of a healthy diet. These results were similar to previous studies, although in isolation. In this sense, different studies showed that metacognitive strategies have been positively related to healthy habits such as physical activity practice (Theodosiou et al., 2008; Liu et al., 2019; Coimbra et al., 2021), similarly, previous studies showed how critical thinking has been positively related to habits related to physical activity practice (Pill and SueSee, 2017). However, engagement in learning has not been found to be related to healthy lifestyle habits, although there are studies that have shown a positive relationship between commitment and motivation toward classes with respect to physical activity and healthy diet (Ulstad et al., 2016; Hastie et al., 2022). In this way, the use of metacognitive strategies, critical thinking and engagement in learning constitute a number of variables that can foster students’ commitment to not only academic achievement, but also greater wellbeing related to their quality of life. In this sense, the most engaged students are those who strategically self-regulate their learning processes, making use of planning strategies, monitoring the completion of their tasks and maintaining intense critical judgment (Sperling et al., 2016; Rigo, 2017), improving their motor skills and ultimately gaining a self-perception of improved health (Sicilia et al., 2014). Furthermore, previous studies have shown that engagement in PE classes is directly related to the adoption of healthy routines outside this context (Jiménez-Castuera et al., 2007), with this commitment and involvement of students being malleable and sensitive to the variables of the context, with critical thinking being an indispensable tool in the transfer of knowledge and its application in problem solving.

Despite the relevance of the results obtained, it is necessary to bear in mind some limitations. Firstly, in terms of methodological issues, as this is a correlational study, it is not possible to determine cause-effect relationships, so the model presented is one possibility, in this case the one that best fits the literature reviewed, as other variables (e.g., motivation, emotions, etc.) could have been included, but due to the complexity of the model it was not possible to do so. It would be interesting for future research to design applied research to explore other possible relationships between the variables considered, with the aim of optimizing the benefits provided by the PE classes.

Finally, this work can help to consolidate a teaching model that allows the reorganization of the teacher’s thinking, generating new educational practices such as gamification and/or game-based learning that help to gain internal coherence in the curriculum itself so that the information to be transmitted to students is close to them, enjoyable and in line with their interests.

## Conclusion

The present study confirms the findings of previous studies (Trigueros et al., 2019b) that support the importance of PE classes in influencing and providing a series of resources and skills that are crucial for the adoption of positive health-related behaviors (Ekblom-Bak et al., 2018). To this end, teacher-generated learning experiences are essential to foster student engagement and thus achieve the academic objectives of the PE subject. In this way, PE should be considered as an area of great importance and interest in the development of values and attitudes through a climate that fosters student learning, involvement and commitment (Errisuriz et al., 2018). This would go some way to resolving the existing gaps in the literature on these relationships, opening up an interesting line of work whose results can be of great use in the field of teaching.

## Data Availability Statement

The datasets generated during and/or analyzed during the current study are not publicly available because we do not have the consent of the study participants but are available from the corresponding author on reasonable request.

## Ethics Statement

The studies involving human participants were reviewed and approved by the Bioethics Committee of the University of Almería (Ref. UALBIO 2020/014) and respected the procedures established by the Declaration of Helsinki. Written informed consent to participate in this study was provided by the participants’ legal guardian/next of kin.

## Author Contributions

All authors listed have made a substantial, direct, and intellectual contribution to the work, and approved it for publication.

## Conflict of Interest

The authors declare that the research was conducted in the absence of any commercial or financial relationships that could be construed as a potential conflict of interest.

## Publisher’s Note

All claims expressed in this article are solely those of the authors and do not necessarily represent those of their affiliated organizations, or those of the publisher, the editors and the reviewers. Any product that may be evaluated in this article, or claim that may be made by its manufacturer, is not guaranteed or endorsed by the publisher.
